# Central nervous system, cranial, and cerebrovascular conditions beyond the Kimonis criteria in patients with Gorlin syndrome: a retrospective cohort study

**DOI:** 10.3389/fneur.2026.1935340

**Published:** 2026-10-06

**Authors:** Yukie Arahata, Katsunori Fujii, Daisuke Sawada, Tomoko Uchida, Takeshi Yaga, Tadashi Shiohama

**Affiliations:** 1 Department of Pediatrics, International University of Health and Welfare Narita Hospital, Narita, Japan; 2 Department of Pediatrics, Graduate School of Medicine, Chiba University, Chiba, Japan

**Keywords:** central nervous system, cerebrovascular disease, cranial malformation, Gorlin syndrome, hedgehog signaling, nevoid basal cell carcinoma syndrome, *PTCH1*, retrospective cohort

## Abstract

**Background:**

Gorlin syndrome is a multisystem developmental and cancer-predisposing disorder commonly caused by germline *PTCH1* variants. The Kimonis clinical criteria capture the canonical phenotype; however, central nervous system (CNS), cranial, and cerebrovascular conditions documented beyond these criteria remain poorly defined.

**Methods:**

We conducted a retrospective descriptive cohort study of 32 patients who fulfilled the Kimonis clinical criteria and received care at either or both hospitals in Japan. Clinical information through March 31, 2026, including sufficiently documented care elsewhere, was reviewed. The exploratory primary descriptive outcome was defined as the presence of at least one documented condition in three categories: structural CNS or cranial conditions, cerebrovascular disorders, or CNS neoplasms other than medulloblastoma. The three categories were considered separately.

**Results:**

A *PTCH1* sequence variant or whole-gene deletion was documented in 24 of 32 patients. Eight patients had 10 documented findings representing eight conditions, including hydrocephalus, Chiari malformation, syringomyelia, moyamoya disease, and cerebral infarction. These findings were identified during routine longitudinal clinical care rather than systematic neuroimaging screening and therefore should not be interpreted as a prevalence estimate. Four patients underwent neurosurgical or cerebrovascular interventions.

**Conclusion:**

Clinically relevant CNS, cranial, and cerebrovascular conditions documented beyond the Kimonis criteria may warrant consideration when new neurological, respiratory, or craniocervical symptoms arise. Awareness of these conditions may inform symptom-directed differential diagnosis and the selection of imaging modalities and anatomical coverage. These exploratory findings do not establish a unified neurological phenotype or causal relationship with Gorlin syndrome and do not support universal neuroimaging surveillance in asymptomatic patients.

## 1 Introduction

Gorlin syndrome, also known as nevoid basal cell carcinoma syndrome, is a multisystem developmental and cancer-predisposing disorder most commonly caused by germline pathogenic variants of *PTCH1* and dysregulation of Hedgehog signaling (1–3). This syndrome is characterized by basal cell carcinomas, odontogenic keratocysts, palmoplantar pits, ectopic intracranial calcification, skeletal abnormalities, and an increased risk of certain tumors. Hedgehog signaling plays a fundamental role in embryonic patterning, organogenesis, and tissue homeostasis, thereby providing a biological basis for the broad phenotype of the disorder (4).

The Kimonis clinical criteria capture the canonical phenotype and were used as the clinical eligibility framework for the present historical cohort. However, contemporary evaluation of Gorlin syndrome increasingly incorporates molecular findings, including *PTCH1* and SUFU, because genotype may influence phenotype and surveillance considerations (5, 6). Accumulating evidence also indicates that patients can present with a broader clinical spectrum that varies according to age and population background (7–9). Japanese cohorts have reported a lower frequency of basal cell carcinoma than that generally described in Western cohorts (8, 10). However, the aggregate spectrum and clinical significance of CNS, cranial, and cerebrovascular manifestations not included in the Kimonis clinical criteria remain incompletely defined.

We began treating Gorlin syndrome in 2000 and reported a case of empty sella in the same year (11). In 2009, we analyzed 25 Japanese patients with Gorlin syndrome and reported that two cases included hydrocephalus (12). In 2012, we conducted a domestic epidemiological survey of Gorlin syndrome in Japan and reported the prevalence of Kimonis’ diagnostic criteria, but we did not investigate central nervous system symptoms (10). Since then, we have encountered central nervous system symptoms more frequently in the treatment of Gorlin syndrome.

In the present study, we retrospectively reviewed 32 patients diagnosed with Gorlin syndrome based on the Kimonis clinical criteria who received care at two tertiary care hospitals in Japan. The aim of this study was to delineate the spectrum and clinical consequences of central nervous system, cranial, and cerebrovascular manifestations not included in the Kimonis clinical criteria used for cohort eligibility. We further consider how awareness of these manifestations may inform symptom-directed differential diagnosis, imaging modalities, and anatomical coverage.

## 2 Methods

### 2.1 Study design, setting, and participants

This was a retrospective descriptive cohort study based primarily on a historical cohort of patients with Gorlin syndrome at Chiba University Hospital, supplemented by eligible patients subsequently identified at International University of Health and Welfare Narita Hospital. The historical Chiba University cohort was assembled through longitudinal clinical care and formed the basis of our previous reports. Additional eligible patients receiving care at Narita Hospital were subsequently added to the cohort. Patients who received care at both institutions were identified as the same individuals and counted only once. The data cutoff date was March 31, 2026, and data were extracted between April and June 2026.

Eligibility for the present analysis required fulfillment of the prespecified Kimonis clinical criteria, defined as at least two major criteria or one major criterion plus at least two minor criteria (8). Molecular confirmation was not required for inclusion. The Kimonis criteria were used as the prespecified clinical eligibility framework for this historical cohort and were not intended to represent a comprehensive contemporary molecular diagnostic framework for Gorlin syndrome. After review of the available medical records and related source documents, 32 unique patients in the assembled cohort met the eligibility criteria and were included in the analysis. Formal sample size calculations were not conducted.

### 2.2 Longitudinal follow-up and data sources

The patients generally attended periodic outpatient follow-ups at one or both of the participating hospitals. During routine visits, clinicians obtained and documented the interval medical history from the preceding visit, including any newly diagnosed conditions, examinations, hospitalizations, surgical procedures, and other treatments provided at the participating hospitals or other medical facilities. The clinical history analyzed was not restricted to investigations or treatments performed at any of the participating hospitals. Neurological diagnoses were verified primarily from referral letters provided by the treating physicians. Radiology reports were additionally available for selected patients, including those with cerebral infarction and moyamoya disease. Original imaging studies were not uniformly available for direct review by the study investigators.

Demographic, clinical, neuroimaging, treatment, outcome, and molecular data were extracted from longitudinal outpatient records, referral letters, external discharge and operative summaries, radiology reports, available neuroimaging studies, pathology reports, and original genetic testing results. Manifestations were considered confirmed when they were supported by an available source document or explicitly documented by a clinician as a confirmed diagnosis in longitudinal medical records. When information differed among the sources, the original diagnostic, imaging, operative, or genetic testing reports were prioritized over any secondary clinical summaries. Patient- or guardian-reported conditions without sufficient diagnostic documentation were excluded from the primary analysis.

### 2.3 Study focus, variables, and outcome definitions

This study comprised a focused secondary analysis of a cohort in which manifestations not included in the Kimonis clinical criteria were previously surveyed across different organ systems. Before detailed source record abstraction for this analysis, the target outcomes were defined as central nervous system, cranial, and cerebrovascular conditions, as these formed a clinically coherent group and included potentially consequential neurological events. No comparative analyses were conducted across different organ systems.

The extracted variables included sex, age at initial cohort compilation, follow-up duration, family history, diagnostic criteria, neurological symptoms, neuroimaging modality, age at diagnosis of each target manifestation, treatment, clinical outcome, and available molecular findings. The follow-up duration was calculated from the first available source record until March 31, 2026. Dedicated cerebrovascular imaging modalities included magnetic resonance angiography, computed tomography (CT) angiography, and catheter angiography.

The primary descriptive outcome was defined, for exploratory purposes, as the presence of at least one documented condition beyond the Kimonis criteria in any of three analytic categories: (1) structural central nervous system or cranial conditions, (2) cerebrovascular disorders, and (3) central nervous system neoplasms other than medulloblastoma. The three categories were considered separately and were not assumed to represent a unified neurological phenotype or to share a common pathophysiological mechanism. Cerebrovascular disorders were retained as a distinct category because of their clinical relevance; their inclusion did not imply a causal relationship with Gorlin syndrome. Medulloblastoma, macrocephaly, ovarian fibroma, and other manifestations included in the Kimonis criteria were not considered primary outcomes. A pineal cyst was described separately because its clinical significance and relationship to Gorlin syndrome were uncertain. Epilepsy, sleep-disordered breathing, and procedures such as shunt placement were considered associated clinical features unless an underlying target diagnosis was documented. Each patient was counted once for the patient-level summary, whereas distinctly named conditions in the same patient were counted separately in the manifestation-level description.

### 2.4 Molecular data

Genetic testing was recommended when clinically appropriate and was performed only after obtaining consent. No additional molecular tests were performed. All available *PTCH1* findings were transcribed from original clinical molecular reports. Molecular data were descriptively classified into four categories: *PTCH1* sequence variant, whole-gene deletion, no *PTCH1* variant detected by testing, and unavailability. When available, cDNA-level descriptions were retained as recorded in the original reports. Testing methods, the use of deletion/duplication analysis, reference transcripts, and reporting standards all varied over time and across patients. Because some historical reports lack sufficient information to allow contemporary reinterpretation, no new pathogenicity classification was assigned for this analysis.

### 2.5 Missing data and bias mitigation

A variable was classified as absent when the available records supported its absence. If no relevant examination was conducted, the variable was classified as unassessed. If the available records were insufficient to determine status, the variable was classified as unknown. Missing values were not imputed and denominators were explicitly reported when they differed from the complete cohort.

To reduce selection bias within the assembled cohort, all patients who met the prespecified eligibility criteria were included. To reduce outcome misclassification, the target manifestations required source document support or explicit clinician documentation, and all primary outcome classifications were reviewed by consensus. Nevertheless, clinical ascertainment reflected routine care, rather than a standardized research imaging protocol. Therefore, the absence of additional neuroimaging was not interpreted as evidence that a structural or cerebrovascular manifestation was absent.

### 2.6 Statistical analysis

Analyses were descriptive. Continuous variables were summarized using medians, interquartile ranges, and ranges, and categorical variables were summarized using counts and percentages. No inferential statistical analyses were performed. Analyses were conducted using Microsoft Excel for Microsoft 365, Version 2607 (Microsoft Corporation, Redmond, WA, USA).

### 2.7 Ethics statement

This retrospective observational study was reviewed and approved by the ethics committees of the International University of Health and Welfare Graduate School of Medicine and Chiba University Graduate School of Medicine (approval numbers 23-Nr-042 and HS202412-01, respectively). Written informed consent regarding participation in this study was provided by the participant’s legal representative/nearest relative. In accordance with the institutional requirements, information about the study was disclosed through an opt-out process. The analysis dataset included only de-identified study identifiers.

## 3 Results

### 3.1 Participant selection and cohort characteristics

The assembled cohort comprised 32 patients who fulfilled the prespecified Kimonis clinical eligibility criteria. Of these, 28 originated from the historical cohort at Chiba University Hospital, and four additional patients were diagnosed at International University of Health and Welfare Narita Hospital. Ten patients originally treated at Chiba University Hospital subsequently continued care at Narita Hospital. Patients who received care at both institutions were identified as the same individuals and counted only once; therefore, there was no duplicate inclusion in the analytic cohort. One patient diagnosed at Narita Hospital underwent genetic testing at Chiba University Hospital.

In 2009, we reported 25 cases of Gorlin syndrome in Japanese patients (12). This included the two cases of hydrocephalus reported here and the cases reported in 2000 (11). In 2012, we analyzed the data including the 25 cases reported in 2009 (10). Since then, seven more cases of Gorlin syndrome have been added, so this time we analyzed central nervous system lesions in 32 cases of Gorlin syndrome.

Following a source record review, all 32 patients met the eligibility criteria and were included in the analysis; no patients were excluded. Sex and age data at initial cohort compilation were available for all patients. The cohort comprised 17 female (53.1%) and 15 male (46.9%) patients. The median age at initial cohort compilation was 19.5 years (interquartile range [IQR], 16.0–38.5; range, 4–66 years). The median follow-up duration was 8.0 years (IQR, 5.0–12.0 years; range, 1.0–20.0 years).

All 32 patients underwent head CT to assess the presence of falx cerebral calcification. Additional brain magnetic resonance imaging (MRI) was conducted in 11 patients (34.4%), while dedicated cerebrovascular imaging was performed in one patient (3.1%). One patient had medulloblastoma, which was classified as a diagnostic manifestation and therefore not counted as a primary outcome (Table 1).

**Table 1:** Demographic, clinical, molecular, and imaging characteristics of the enrolled cohort.

Characteristic	Value
Participants	32
Female sex, *n* (%)	17 (53.1)
Male sex, *n* (%)	15 (46.9)
Affected first-degree relative, *n* (%)	16 (50.0)
Follow-up duration, y, median (IQR; range)	8.0 (5.0–12.0; 1.0–20.0)
Neuroimaging	
Head CT, *n* (%)	32 (100)
Additional brain MRI, *n* (%)	11 (34.4)
Dedicated cerebrovascular imaging, *n* (%)	1 (3.1)
Major diagnostic manifestations	
Basal cell carcinoma, *n* (%)	8 (25.0)
Palmoplantar pits, *n* (%)	27 (84.4)
Odontogenic keratocyst, *n* (%)	25 (78.1)
Rib abnormality, *n* (%)	15 (46.9)
Falx cerebri calcification, *n* (%)	24 (75.0)
Medulloblastoma, *n* (%)	1 (3.1)
*PTCH1* molecular result	
Sequence variant (23) or whole-gene deletion (1) documented, *n* (%)	24 (75.0)
No variant detected by the testing performed, *n* (%)	2 (6.3)
Molecular findings unavailable, *n* (%)	6 (18.8)
CNS, cranial, and cerebrovascular outcomes	
Primary CNS, cranial, or cerebrovascular manifestation, patients, *n*	8
Recorded primary manifestations, *n*	10
Neurosurgical or cerebrovascular intervention among primary-outcome patients, *n*/*N* (%)	4/8 (50.0)
Additional CNS findings analyzed separately	
Potentially incidental pineal cyst, *n* (%)	1 (3.1)

Values are expressed as counts and percentages, unless otherwise stated. Percentages use 32 as the denominator, unless a different denominator is shown. Dedicated cerebrovascular imaging modalities include magnetic resonance angiography, computed tomography angiography, and catheter angiography. Molecular findings were reported as recorded in available source reports and did not constitute contemporary reclassification. CNS, central nervous system; CT, computed tomography; MRI, magnetic resonance imaging.

Of the 32 patients, *PTCH1* variant analysis was performed on 26, identifying mutations in 23 and finding a whole-gene deletion in 1 patient. Genetic analysis was not performed on 6 patients because consent was not obtained (Table 1).

### 3.2 Central nervous system, cranial, and cerebrovascular conditions

By the data cutoff date, eight patients had at least one documented condition included in the exploratory patient-level summary. Patient-level fulfillment of the Kimonis criteria is summarized in Supplementary Table 1. These patients had 10 recorded manifestations representing eight conditions (Figure 1; Table 2). Because additional brain MRI and dedicated cerebrovascular imaging were not performed uniformly, these findings reflect conditions documented during routine longitudinal clinical care rather than a prevalence estimate based on systematic neuroimaging; clinically silent conditions may have remained undetected. The three categories were reported separately.

**Figure 1:**
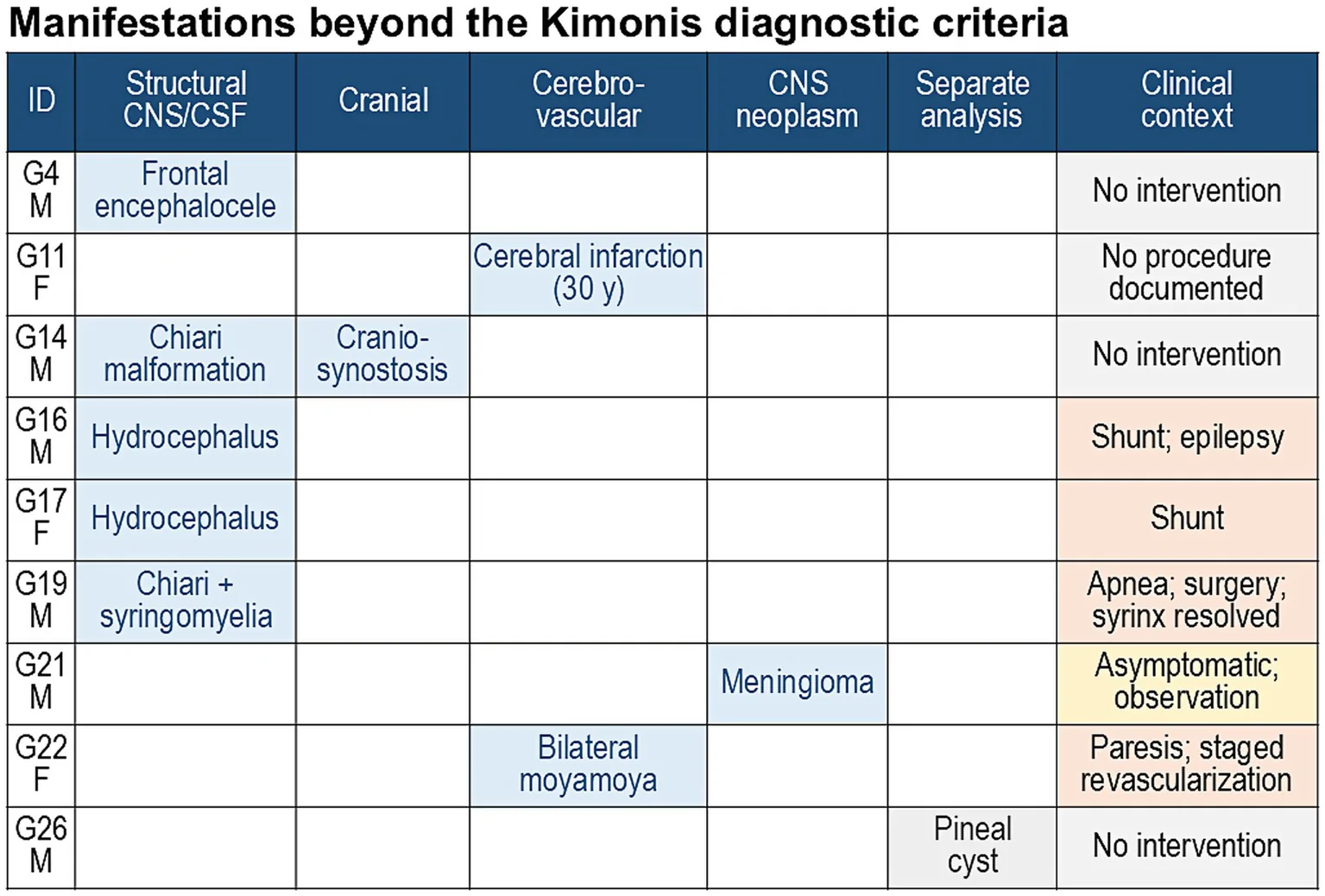
Documented conditions not included in the Kimonis clinical criteria used for cohort eligibility. Blue cells indicate conditions included in the exploratory descriptive summary. The gray cell indicates a pineal cyst, which was analyzed separately. Empty cells do not distinguish between conditions that were not assessed and conditions that were not documented in the available records. Because MRI and dedicated cerebrovascular imaging were not performed uniformly, empty cells should not be interpreted as confirmed absence. CNS, central nervous system; CSF, cerebrospinal fluid; MRI, magnetic resonance imaging; y, years.

**Table 2:** Patient-level central nervous system, cranial, and cerebrovascular manifestations not included in the Kimonis clinical criteria used for cohort eligibility.

Study ID	Sex; age	*PTCH1* finding as recorded	Category	Manifestation and diagnostic context	Clinical course/evidence	Management and outcome
G4	M; 16 y	c.3130_3131dupGC	Cranial/structural CNS	Frontal encephalocele; CT documented	Diagnostic age and presenting symptoms were not documented in the available summary	No neurosurgical intervention was documented through the data-cutoff date
G11	F; 40 y	c.3527_3528delCT	Cerebrovascular	Cerebral infarction at 30 y; diagnosis supported by the available radiology report	Anatomical distribution and presumed mechanism could not be reconstructed from the available source documentation.	No neurosurgical or revascularization procedure was documented
G14	M; 20 y	c.1847+1G>A	Cranial/structural CNS	Chiari malformation with craniosynostosis; CT documented	Presenting symptoms and diagnostic age were not documented in the available summary	No neurosurgical intervention was documented through the data-cutoff date
G16	M; 19 y	c.328G>C	Structural CNS/CSF	Hydrocephalus requiring shunt placement; epilepsy documented	Hydrocephalus and associated epilepsy were recorded during longitudinal follow-up	Shunt placement; follow-up information available
G17	F; 10 y	c.2668delA	Structural CNS/CSF	Hydrocephalus requiring shunt placement; CT documented	Hydrocephalus was recorded during longitudinal follow-up	Shunt placement; follow-up information available
G19	M; 12 y	c.2668delA	Craniocervical/spinal CNS	Chiari malformation with syringomyelia and sleep-disordered breathing; CT followed by MRI	At approximately 9 y, sleep-disordered breathing prompted evaluation; CT showed ventricular enlargement and MRI showed Chiari malformation with syringomyelia	Surgery at approximately 9 y; syrinx no longer evident by 12 y
G21	M; 56 y	Whole-gene deletion	CNS neoplasm	Mild asymptomatic meningioma; MRI documented	Detected without attributable symptoms	Managed by observation
G22	F; 40 y	No *PTCH1* variant detected	Cerebrovascular	Bilateral moyamoya disease presenting at approximately 6 y with paresis while inflating a balloon; diagnosis supported by the available referral documentation and radiology report	Dedicated cerebrovascular imaging and subsequent treatment were documented; however, the original vascular images and the complete evaluation for secondary causes were not available for study review.	Staged indirect revascularization at 7 and 8 y; follow-up information available
G26	M; 22 y	c.1519delC	Potentially incidental finding	Pineal cyst; MRI documented	Age and reason for detection were not documented	No intervention documented; excluded from the primary outcome

Diagnoses and treatments conducted at other institutions were included if supported by available source documents or explicit clinician notes. Neurological diagnoses were verified primarily from referral letters provided by the treating physicians. Radiology reports were additionally available for the patients with cerebral infarction and moyamoya disease. Original imaging studies were not uniformly available for direct review by the study investigators. CNS, central nervous system; CSF, cerebrospinal fluid; CT, computed tomography; F, female; M, male; MRI, magnetic resonance imaging.

Structural central nervous system or cranial conditions, cerebrovascular disorders, and central nervous system neoplasm were documented in five, two, and one patients, respectively. Chiari malformation and hydrocephalus were documented in two patients each; frontal encephalocele, craniosynostosis, syringomyelia, cerebral infarction, bilateral moyamoya disease, and meningioma were documented in one patient each.

Seven of eight patients included in the exploratory patient-level summary had documented *PTCH1* sequence variants or whole-gene deletions; however, the patient with moyamoya disease had no *PTCH1* variants detected in the performed tests. A pineal cyst was documented in one additional patient and analyzed separately as it was not included in the primary outcome.

### 3.3 Clinical presentation, treatment, and outcomes

Neurosurgical or cerebrovascular interventions were documented in four of the eight patients with documented conditions (50.0%). Two patients underwent shunt placement because of hydrocephalus. One patient underwent an evaluation for sleep-disordered breathing at approximately 9 years of age. CT revealed ventricular enlargement while subsequent MRI revealed a Chiari malformation with syringomyelia. Surgery was performed at approximately 9 years of age, and the syrinx was no longer evident at 12 years of age (Figure 2). Another patient developed paresis while inflating a balloon at approximately 6 years of age and was subsequently diagnosed with bilateral moyamoya disease. Staged indirect revascularization procedures were conducted at 7 and 8 years of age.

**Figure 2:**
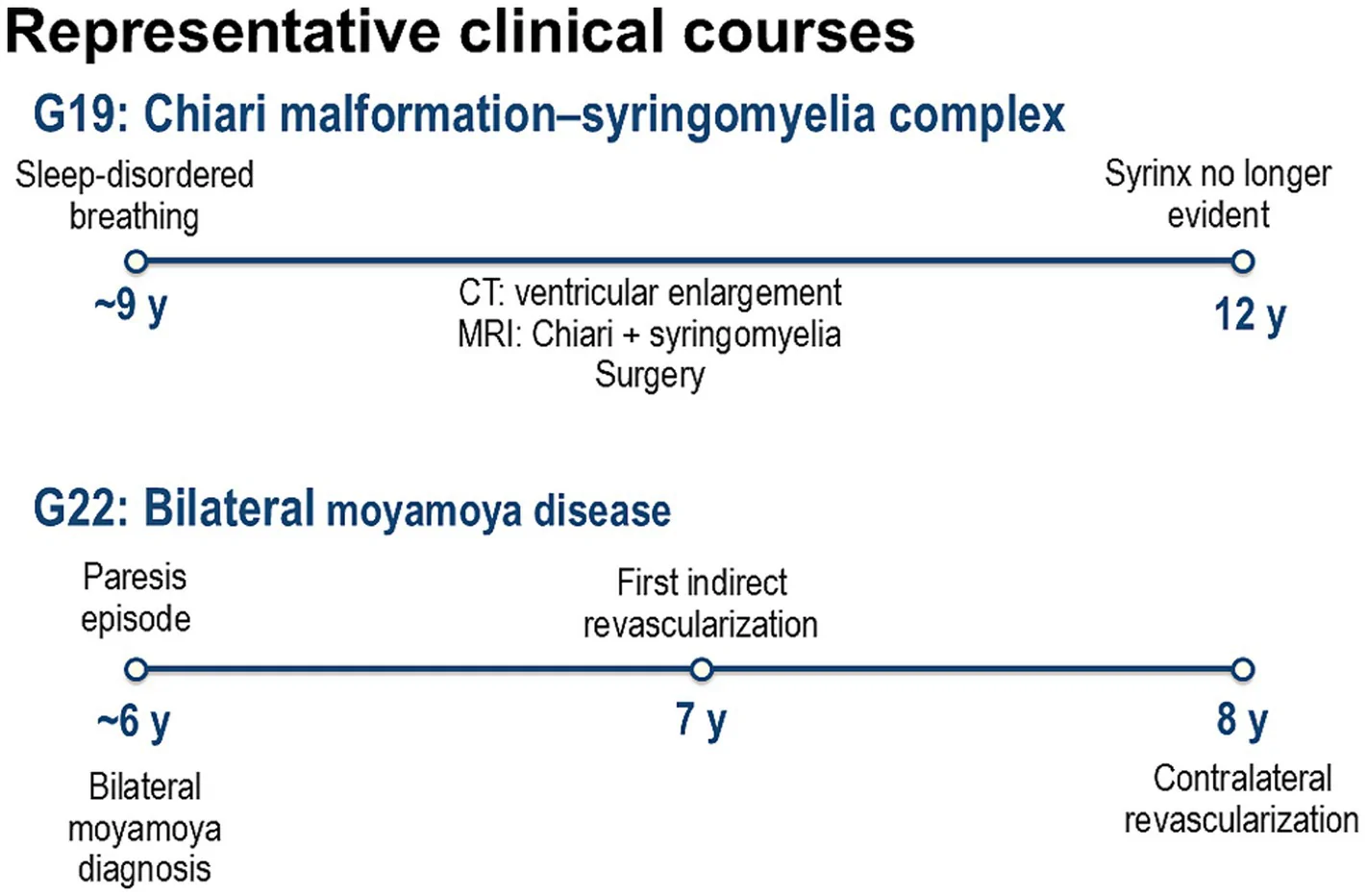
Representative clinical courses of patients G19 and G22. In G19, evaluation for sleep-disordered breathing at approximately 9 years of age led to CT examination, which revealed ventricular enlargement. Subsequent MRI demonstrated Chiari malformation with syringomyelia. Surgical treatment was performed during the same period, and the syrinx was no longer evident at 12 years of age. In G22, an episode of paresis at approximately 6 years of age led to the diagnosis of bilateral moyamoya disease. Staged indirect revascularization procedures were performed at 7 and 8 years of age. CT, computed tomography; MRI, magnetic resonance imaging; y, years.

Among the remaining four patients, one asymptomatic meningioma was managed through observation. No neurosurgical or cerebrovascular interventions were documented for frontal encephaloceles, cerebral infarctions, or Chiari malformations with craniosynostosis. In this patient, the cerebral infarction occurred at 30 years of age, although its anatomical distribution and presumed mechanism were not documented. Further details regarding manifestations, treatments, and outcomes are presented in Table 2.

Follow-up information was available for all eight patients included in the exploratory patient-level summary.

## 4 Discussion

This study documented a heterogeneous set of clinically relevant central nervous system, cranial, and cerebrovascular conditions beyond the Kimonis criteria in patients diagnosed with Gorlin syndrome. Eight patients had at least one condition included in the exploratory patient-level summary, corresponding to 10 recorded manifestations, and four underwent neurosurgical or cerebrovascular interventions. This summary was used only to describe documented conditions and should not be interpreted as evidence of a unified neurological phenotype. The structural CNS or cranial, cerebrovascular, and neoplastic categories were considered separately because they differ substantially in origin, pathophysiology, age of occurrence, and clinical significance.

Overseas cohorts have also documented CNS and cranial findings of basal cell nevus syndrome in Gorlin syndrome cohorts. For example, one nationwide Norwegian cohort of 103 individuals recorded cases of hydrocephalus, intracranial tumors, epilepsy, intracranial cysts, Chiari malformation, craniosynostosis, and stroke (13). Presentations in our cohort overlapped with these reports in several respects, including hydrocephalus, Chiari malformation, craniosynostosis, meningioma, and cerebral infarction. However, the observed spectrum also included frontal encephalocele and bilateral moyamoya disease, which were not prominent in an overseas summary. This contrast suggests that the non-criteria neurological spectrum encountered in clinical practice may not be fully captured by the previously emphasized manifestations.

Population-level differences have been described in the canonical manifestations of Gorlin syndrome. In particular, one Japanese cohort (10) reported a lower frequency of basal cell carcinoma than that generally described in Western cohorts (8). This context is relevant when considering manifestations beyond the diagnostic criteria as the observed clinical spectrum may be shaped by the genetic background, age structure, referral pathways, imaging practices, and healthcare settings. The present cohort does not answer this comparative question, but provides patient-level data that may support future standardized international comparisons.

The primary contribution of this study is the detailed patient-level characterization of CNS, cranial, and cerebrovascular manifestations not included in the Kimonis clinical criteria used for cohort eligibility. We provide information on presenting symptoms, diagnostic context, interventions, and clinical outcomes. This patient-level characterization is clinically relevant because some manifestations were not merely incidental imaging findings but were associated with symptoms or events requiring intervention. Evaluation of CNS and cranial manifestations as part of the Gorlin syndrome spectrum is biologically and radiologically plausible. Shiohama et al. previously demonstrated group-level brain morphometric differences in affected children, including enlargement of the cerebral and cerebellar structures, ventricular enlargement, and corpus callosum thinning (14). Hedgehog signaling also regulates neural patterning, progenitor cell proliferation, cerebellar development, and craniofacial morphogenesis (4). Therefore, *PTCH1*-dependent dysregulation may influence the brain and cranial development. However, the observed conditions were heterogeneous and the present data did not support any single causal mechanism for all manifestations.

The clinical value of the two illustrative cases lies in showing why symptom-directed neurological evaluation may be important, not in establishing a new imaging algorithm for Gorlin syndrome. In G19, evaluation for sleep-disordered breathing led to CT, which revealed ventricular enlargement; subsequent MRI demonstrated Chiari malformation with syringomyelia. This course suggests that, when sleep-disordered breathing is atypical, progressive, accompanied by neurological findings, or associated with another imaging abnormality, targeted imaging of the craniocervical junction and spinal cord may be considered. In G22, a focal neurological event led to dedicated vascular imaging and the diagnosis of bilateral moyamoya disease. The diagnosis was supported by the available referral documentation and radiology report. However, the original vascular images and the complete diagnostic evaluation for secondary causes were not available for study review. Accordingly, the etiology of the moyamoya disease and its potential relationship to Gorlin syndrome cannot be established from the present data. The referring physician noted that the meningioma in G21 occurred adjacent to the falx cerebri in the parietal region. These observations are hypothesis-generating only and do not support routine brain MRI or cerebrovascular imaging in asymptomatic patients. Existing clinical management recommendations for Gorlin syndrome state that, in children with a *PTCH1* pathogenic variant, brain MRI should be considered when clinical symptoms or abnormal psychomotor development are present, whereas routine MRI is not indicated (6). Although these recommendations were developed primarily in the context of medulloblastoma surveillance, they support a symptom-directed rather than routine brain MRI approach in *PTCH1*-related disease. Our observations are consistent with this approach and should be viewed as complementary to, rather than as a basis for modifying, existing genotype-specific surveillance recommendations.

The Kimonis clinical criteria were used as the prespecified eligibility framework for this historical cohort; however, they should not be regarded as a substitute for contemporary molecular diagnosis. Genotype-specific differences have been reported in Gorlin syndrome, particularly between *PTCH1*- and SUFU-associated disease, with implications for tumor risk and surveillance (5, 6). One patient in the present cohort had medulloblastoma; this manifestation was excluded from the exploratory primary outcome because it is included in the Kimonis clinical criteria. Because molecular testing in this retrospective cohort was performed over many years using heterogeneous methods, systematic contemporary molecular reclassification was not possible. Contemporary clinical assessment should therefore incorporate molecular findings and genotype-informed surveillance when available. All patients underwent head CT as part of the historical clinical practice of this cohort for assessment of falx cerebri calcification; this should not be interpreted as a recommendation for repeated CT-based surveillance. Additional brain MRI was conducted in 11 patients, and dedicated cerebrovascular imaging was performed in one patient. Imaging beyond CT was performed according to clinical indications rather than a standardized research protocol. Accordingly, symptom-driven testing may have increased detection among symptomatic patients, whereas clinically silent abnormalities may have remained unrecognized in patients who did not undergo additional imaging. The number of patients with documented conditions therefore represents only the documented burden during routine clinical care, and the true frequency of these conditions within the cohort cannot be determined from the present data.

This study has several limitations. Firstly, this retrospective study included only 32 patients from two tertiary-care hospitals, which may limit the generalizability of the findings. External source documents were not uniformly available, original images were not uniformly reviewed, and molecular testing methods varied over time. Furthermore, the focus of the organ system was selected following an earlier broad review of manifestations in the cohort, which may have introduced an outcome selection bias. The apparent prominence of central nervous system, cranial, and cerebrovascular findings should not be interpreted as evidence that they are more frequent than non-criteria findings in other organ systems. These limitations preclude population prevalence estimates, causal attribution, and genotype–phenotype conclusions and restrict generalizability to similar patients receiving longitudinal multidisciplinary care in specialized settings. Prospective multicenter studies using standardized phenotyping and imaging protocols are required to determine manifestations that are enriched and clinically relevant.

In conclusion, clinically relevant central nervous system, cranial, and cerebrovascular conditions not included in the Kimonis clinical criteria were documented during longitudinal care of patients diagnosed with Gorlin syndrome. Some manifestations were symptomatic and required intervention. These exploratory observations suggest that such conditions may warrant consideration during symptom-directed evaluation but do not establish that all reported conditions form a unified neurological phenotype or are caused by Gorlin syndrome. Differential diagnosis and imaging should be tailored to the presenting symptoms and clinical context. These findings do not establish population prevalence, causality, genotype–phenotype relationships, or a rationale for universal neuroimaging surveillance in asymptomatic patients.

## Data Availability

The datasets analyzed in this study contained clinical information from individuals with a rare disorder and were not publicly available because of the risk of re-identification. A de-identified minimum dataset may be made available from the corresponding author upon reasonable request, subject to applicable ethical and institutional data-governance requirements.
